# Evidence summary of lifestyle interventions in adults with metabolic dysfunction-associated steatotic liver disease

**DOI:** 10.3389/fnut.2024.1421386

**Published:** 2025-01-06

**Authors:** Mei-jing Chen, Ying Chen, Jin-qing Lin, Rong Hu, Dun Liu, Jing-yi Chen, Ka Li, Xiao-ying Jiang

**Affiliations:** ^1^School of Nursing, Fujian Medical University, Fuzhou, China; ^2^Department of Nursing, Xiamen Medical College, Xiamen, China; ^3^Department of Gastroenterology, Fuzhou Second General Hospital, Fuzhou, China; ^4^West China School of Nursing, Sichuan University, Chengdu, China

**Keywords:** evidence-based nursing, evidence summary, lifestyle, intervention, metabolic dysfunction-associate fatty liver disease, non-alcoholic fatty liver disease

## Abstract

**Objective:**

In this study, our objective was to provide practice recommendations by thoroughly examining lifestyle interventions for adults diagnosed with metabolic dysfunction-associated steatotic liver disease (MASLD). This was achieved through a systematic review of the literature, specifically focusing on lifestyle modification interventions in adults with MASLD.

**Methods:**

The PIPOST (Population, Intervention, Professional, Outcome, Setting, and Type of evidence) framework was used to identify the questions for summarizing evidence. Utilizing the 6S model for the hierarchy of evidence, a computerized search was conducted to retrieve articles pertaining to lifestyle interventions for adults with MASLD from websites such as the UpToDate Clinical Advisor, BMJ Best Practice, JBI Library, Cochrane Library, International Guidelines Library, and PubMed, among others. The available research included clinical decisions, clinical practice guidelines, evidence summaries, systematic evaluation, expert consensus, and expert opinions. Two researchers independently evaluated the methodology of the studies, and evidence was subsequently extracted and grouped thematically. Our review encompassed publications from January 2018 to March 2023.

**Results:**

A total of 26 publications were identified for the final review, consisting of seven guidelines, nine systematic evaluations, and 10 expert consensuses/opinions. From these sources, we derived six themes, 28 pieces of evidence: intervention modalities, diet management, exercise management, weight loss management, personalized management, and multidisciplinary collaboration.

**Conclusion:**

In the management of adults with MASLD, healthcare professionals should embrace a multidisciplinary team approach, adhere to the best available evidence, and develop structured and personalized interventions based on the best evidence for lifestyle modifications.

## 1 Introduction

Metabolic dysfunction-associated steatotic liver disease (MASLD) (1) is the updated term to describe steatotic liver disease associated with metabolic syndrome. This nomenclature was chosen to replace non-alcoholic fatty liver disease (NAFLD) and metabolic-associated fatty liver disease (MAFLD) (2, 3). MASLD stands as the most prevalent chronic liver condition globally, boasting a staggering 30% prevalence rate (4). This figure continues to rise annually, underscoring the urgency of addressing this disease as a significant public health concern (5). The escalating incidence of MASLD has been linked to unhealthy lifestyle factors, including sedentary behavior, high-calorie diet intake, and poor dietary balance (6). To date, there are no drugs that have been approved by the Food and Drug Administration for the treatment of MASLD, and lifestyle interventions, such as diet and exercise are considered the cornerstone of treatment for patients with MASLD (7). Multiple dietary patterns such as hypocaloric diet (8), Mediterranean diet (9, 10), and intermittent fasting (11) are adopted in the treatment of patients with MASLD, and all of these dietary strategies have shown evidence of benefit in reducing liver fat and inflammation. Physical activity may also provide significant clinical benefits for patients with MASLD/NAFLD (12).

Literature on MASLD and NAFLD in both Chinese and global contexts includes treatment guidelines, systematic reviews, and expert consensuses. However, the existing evidence pertaining to lifestyle interventions is not concise, and the content is not suitable for widespread dissemination. “There is a notable absence of standardized specific measures, focused evidence extraction on the theme of lifestyle interventions, and concise, user-friendly lifestyle practice guidelines for medical professionals and patients dealing with MASLD. Therefore, in this review, we systematically retrieved, extracted, and summarized high-level evidence-based data from both Chinese and international sources. Our objective was to formulate practice recommendations specifically centered around lifestyle interventions for adults diagnosed with MASLD. This study is intended to inspire healthcare professionals to actively engage in lifestyle interventions for adults with MASLD, serving as a valuable evidence-based resource for researchers aiming to develop well-structured lifestyle intervention programs tailored to this population.

## 2 Materials and methods

### 2.1 Criteria for summarizing evidence

We utilized the PIPOST (Table 1) model to identify specific questions for evidence summarization in this study (13).

**Table 1:** Research methods.

Item	Description
Criteria for summarizing evidence	PIPOST model (13). P (population): the target study population of evidence application is adult patients with MAFLD, MASLD aged ≥18 years. I (intervention): the intervention measures that were lifestyle interventions or related to lifestyle management. P (professional): the professionals who applied evidence are specifically clinicians, nurses, and healthcare professionals. O (outcome): the outcomes included metabolism-related indicators (liver enzymes, lipids, and insulin resistance), liver fat content, and weight change. S (setting): the evidence applied in hospitals, healthcare facilities, and communities. T (type of evidence): the types of evidence are thematic evidence summaries (including clinical decisions, practice recommendations, and evidence summaries), clinical practice guidelines, systematic reviews, expert consensuses, and expert opinions.
Inclusion criteria	studies that were based on the study population as described above; the study content consisted of lifestyle interventions or lifestyle management such as diet, exercise, and weight loss; the type of study was as described above; the language of publication was Chinese and English.
Exclusion criteria	studies on patients with MAFLDMASLD who also had extrahepatic diseases such as renal disease and cardiovascular disease; non-availability of the full text of the article; translations of foreign guidelines, guideline interpretations, duplicate publications, or updated versions; the document failed the literature quality evaluation (guidelines with an evaluation level of C, or the number of items of systematic review, expert consensuses, and expert opinions evaluation ≥50% was “no” or ≥80% was “unclear”) (14).
Evidence-based information databases	UpToDate Clinical Advisor, BMJ Best Practice, JBI Library, Cochrane Library, Guidelines International Network, National Guideline Clearinghouse, the National Institute for Health and Care Excellence, Scottish Intercollegiate Guidelines Network, Registered Nurses’ Association of Ontario, Embase, and the China Medical Pulse Guidelines Network
Other comprehensive databases	PubMed, the Cumulative Index to Nursing and Allied Health Literature, the China National Knowledge Infrastructure, Wanfang, VIP, and the China Biology Medicine disc, as well as the websites of the American Association for the Study of Liver Diseases and the Journal of Clinical Hepatology
Chinese search keywords, English search keywords	“metabolic-associated fatty liver disease,” “non-alcoholic fatty liver disease,” “lifestyle,” “diet” “exercise” “physical activity,” and “weight loss.”
Published time	Between January 2018 and March 2023
Tools for quality evaluation of guidelines	Appraisal of Guidelines for Research and Evaluation Instrument (AGREE) II (16)
Tools for quality evaluation of systematic reviews with meta-analysis	AMSTAR (A MeaSurement Tool to Assess systematic Reviews)-2 (17)
Tools for quality evaluation of systematic reviews, expert consensuses, and expert opinions without meta-analysis	Evidence-Based Healthcare (EBHC) (18) tool of the Joanna Briggs Institute (JBI)

P (population): The target study population for evidence application consisted of adult patients with MASLD aged 18 years or older.

I (intervention): The intervention measures were focused on lifestyle interventions or those related to lifestyle management.

P (professional): The professionals who applied the evidence were primarily clinicians, nurses, and healthcare professionals.

O (outcome): The outcomes encompassed metabolism-related indicators (such as liver enzymes, lipids, and insulin resistance), liver fat content, and changes in weight.

S (setting): The evidence was applied in various settings, including hospitals, healthcare facilities, and communities.

T (type of evidence): The types of evidence encompassed thematic evidence summaries (comprising clinical decisions, practice recommendations, and evidence summaries), clinical practice guidelines, systematic reviews, expert consensuses, and expert opinions.

This project was registered with the Center for Evidence-Based Nursing, Fudan University (No. ES20231902).

### 2.2 Inclusion and exclusion criteria

The inclusion criteria were as follows: (i) studies that were based on the study population as described above; (ii) the study content consisted of lifestyle interventions or lifestyle management such as diet, exercise, and weight loss; (iii) the type of study was as described above; (iv) the language of publication was Chinese or English (Table 1).

The following were the exclusion criteria: (i) studies on patients with MASLD who also had extrahepatic diseases such as renal disease and cardiovascular disease; (ii) non-availability of the full text of the article; (iii) translations of guidelines that were originally not in English or Chinese, guideline interpretations, duplicate publications, or updated versions; (iv) the document failed the literature quality evaluation (guidelines with an evaluation level of C, or the number of items of systematic review, expert consensuses, and expert opinions evaluation ≥50% was “no” or ≥80% was “unclear”) (Table 1) (14).

### 2.3 Search strategies

Based on the “6S” model of evidence resources (15), we adopted a computerized top-down search of evidence-based information databases such as the UpToDate Clinical Advisor, BMJ Best Practice, JBI Library, Cochrane Library, Guidelines International Network, National Guideline Clearinghouse, the National Institute for Health and Care Excellence, Scottish Intercollegiate Guidelines Network, Registered Nurses’ Association of Ontario, Embase, and the China Medical Pulse Guidelines Network. We also additionally searched other comprehensive databases, including PubMed, the Cumulative Index to Nursing and Allied Health Literature, the China National Knowledge Infrastructure, Wanfang, VIP, and the China Biology Medicine disc, as well as the websites of the American Association for the Study of Liver Diseases and the Journal of Clinical Hepatology (Table 1).

The Chinese search keywords we used were “metabolic-associated fatty liver disease,” “non-alcoholic fatty liver disease,” “lifestyle,” “diet,” “exercise,” and “weight loss.” The English search keywords included “metabolic-associated fatty liver disease,” “non-alcoholic fatty liver disease,” “lifestyle,” “diet,” “exercise,” “physical activity,” and “weight loss.” The search strategy is shown in Appendix 1. We searched for articles published between January 2018 and March 2023 (Table 1).

### 2.4 Criteria and process for evidence quality evaluation

We used the appropriate tools for quality evaluation based on the type of evidence. (1) For quality evaluation of guidelines, we used the Appraisal of Guidelines for Research and Evaluation Instrument (AGREE) II (Table 1) (16), comprising six domains and 23 items. Each item was evaluated on a scale of 1–7, and the quality score for each domain was obtained. The overall score was then calculated by summing all the domains. The quality of the guidelines was classified into three levels: grade A when all six domain scores were ≥60%; grade B when scores of ≥3 domains were 30–60%; and grade C when scores of >3 domains were <30%.

(2) For evaluating the quality of systematic reviews with meta-analysis, we used the AMSTAR (A MeaSurement Tool to Assess systematic Reviews)-2 (Table 1) (17), which consists of 16 items, of which the responses to seven key items (items 2, 4, 7, 9, 10, 11, 13, and 15) were “yes,” “partially yes,” and “no.” The quality of the evaluation was classified as “high,” “moderate,” “low,” and “critically low.”

(3) We used the Evidence-Based Healthcare (EBHC) (18) tool of the Joanna Briggs Institute (Table 1) (JBI) to evaluate the quality of systematic reviews, expert consensuses, and expert opinions without meta-analysis. This tool for evaluating systematic reviews and expert consensuses consists of 11 and 6 items, respectively, with the responses being “yes,” “no,” “unclear,” and “not applicable.”

Two researchers systematically trained in evidence-based methods separately evaluated the quality of the included literature, and in the case of conflicting evaluations, a third investigator participated in the discussion to arrive at a consensus.

### 2.5 Extraction, summary, and grading of evidence

Two researchers independently reviewed each article in the cited literature and collected evidence from it based on the theme. In cases where the conclusions and evidence of different sources were inconsistent, evidence was extracted following the criteria of priority of high-quality evidence, priority of recent evidence, and priority of evidence-based information. All the selected clinical practice guidelines, recommended practices, and evidence summaries were summarized with their original grading system (19). The evidence of the guidelines was graded using the Grading of Recommendations, Assessment, Development, and Evaluations (GRADE) system (20), and the levels of evidence were classified as “high (A),” “medium (B),” “low (C),” and “very low (D),” with the recommendation grade as “strong recommendation (1)” and “weak recommendation (2).” For evidence without a grading system, the 2014 version of the JBI evidence pre-grading and evidence recommendation level framework was used (21), and evidence was categorized into levels 1–5 and the recommendation was determined as grade A (strong recommendation) or grade B (weak recommendation) in combination with the JBI feasibility, appropriateness, meaningfulness, and effectiveness (FAME) approach and the JBI recommendation grades.

## 3 Results

### 3.1 Results of literature retrieval

**Figure 1:**
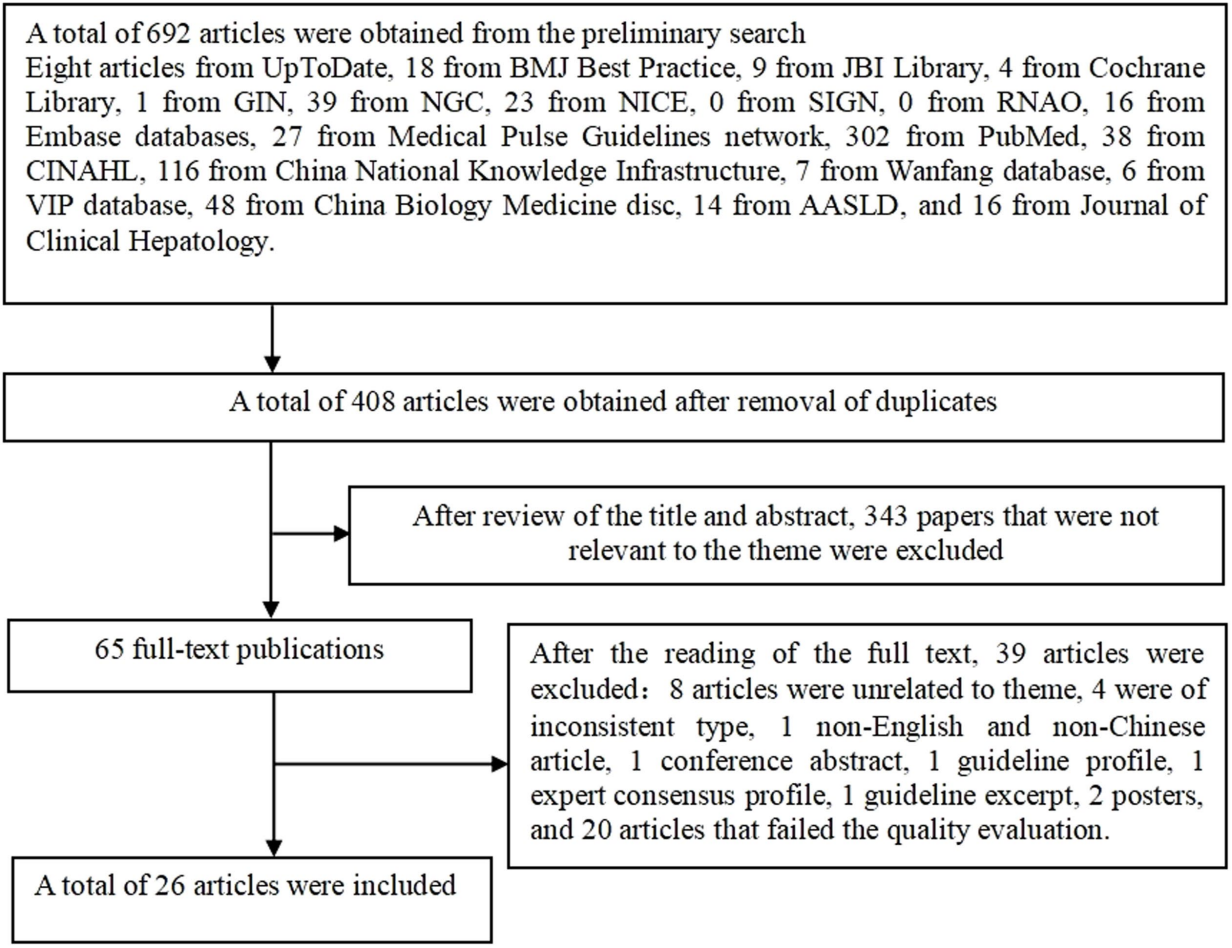
Flow chart of literature identified for the systematic review.

A total of 692 articles were retrieved and finally, 26 articles were included in the review after layer-by-layer screening. The literature screening process is shown in Figure 1.

### 3.2 Profile of the reviewed literature

This review included 26 publications, comprising seven guidelines (22–28), nine systematic evaluations with meta-analyses (29–37), six expert consensuses (38–43), and four expert opinions (44–47). The details of the publications is shown in Table 2.

**Table 2:** Characteristics of included studies.

Included articles	Year of publication (year)	Source of the article	Type of evidence	Theme
Cusi et al. (24)	2022	BMJ and Journal of Clinical Hepatology	Guidelines	Clinical Endocrinology: Clinical Practice Guidelines for the Diagnosis and Management of Nonalcoholic Fatty Liver Disease in Primary Care and Endocrinology Clinical Settings
Aller et al. (23)	2018	Journal of Clinical Hepatology	Guidelines	Management of non-alcoholic fatty liver disease (NAFLD): Clinical practice guideline
Kang et al. (26)	2021	BMJ	Guidelines	KASL clinical practice guidelines: Management of nonalcoholic fatty liver disease
Eslam et al. (25)	2020	Journal of Clinical Hepatology	Guidelines	The Asian Pacific Association for the Study of the Liver: clinical practice guidelines for the diagnosis and management of metabolic associated fatty liver disease
AISF, SID, SIO (22)	2021	Journal of Clinical Hepatology	Guidelines	Non-alcoholic fatty liver disease in adults 2021: A clinical practice guideline of the Italian Association for the Study of the Liver (AISF), the Italian Society of Diabetology (SID) and the Italian Society of Obesity (SIO)
Fouad et al. (28)	2022	PubMed	Guidelines	The Egyptian clinical practice guidelines for the diagnosis and management of metabolic associated fatty liver disease
Fan et al. Chinese Society of Hepatology, National Workshop on Fatty Liver and Alcoholic Liver Disease (27)	2018	China National Knowledge Infrastructure	Guidelines	Guidelines for the prevention and treatment of nonalcoholic fatty liver disease (2018 update)
Chai et al. (30)	2023	UpToDate	Systematic review^*^	Effects of lifestyle intervention on adults with metabolic associated fatty liver disease
Garcêz et al. (33)	2021	PubMed	Systematic review^*^	Effect of dietary carbohydrate and lipid modification on clinical and anthropometric parameters in nonalcoholic fatty liver disease
Reddy et al. (35)	2019	PubMed	Systematic review	Effect of dietary intervention on inflammatory markers in patients with nonalcoholic fatty liver disease
Xiong et al. (37)	2021	PubMed	Meta-analysis	Effect of different exercise methods on non-alcoholic fatty liver disease
Hong et al. (34)	2022	PubMed	Systematic review^*^	Effect of exercise training on serum transaminases in patients with nonalcoholic fatty liver disease
Fu et al. (32)	2022	PubMed	Systematic review^**^	Efficacy of aerobic and resistance exercises in improving visceral adipose in patients with non-alcoholic fatty liver
Słomko et al. (36)	2021	PubMed	Systematic review^**^	Evidence-based aerobic exercise training in metabolic-associated fatty liver disease
Fernández et al. (31)	2022	PubMed	Systematic review^*^	Lifestyle changes in patients with non-alcoholic fatty liver disease
Babu et al. (29)	2021	PubMed	Systematic review^*^	Positive effects of exercise intervention without weight loss and dietary changes in NAFLD-related clinical parameters
Rinella et al. (41)	2023	Journal of Clinical Hepatology	Expert consensus	AASLD Practice Guidance on the clinical assessment and management of nonalcoholic fatty liver disease
Chan et al. (43)	2022	PubMed	Expert consensus	Malaysian Society of Gastroenterology and Hepatology consensus statement on metabolic dysfunction-associated fatty liver disease
Bernal-Reyes et al. (42)	2019	PubMed	Expert consensus	The Mexican consensus on nonalcoholic fatty liver disease
Alswat et al. (40)	2019	PubMed	Expert consensus	Position statement on the diagnosis and management of non-alcoholic fatty liver disease
Lazarus et al. (38)	2021	Journal of Clinical Hepatology	Expert consensus	Advancing the global public health agenda for NAFLD: a consensus statement
Chinese Society of Endocrinology (39)	2018	Medical Pulse Guidelines network	Expert consensus	Consensus for diagnosis and treatment of nonalcoholic fatty liver diseases and metabolic disorders (2nd Edition)
Kamada et al. (47)	2021	PubMed	Expert opinion	Clinical practice advice on lifestyle modification in the management of nonalcoholic fatty liver disease in Japan
George et al. (46)	2018	Embase	Expert opinion	Practical dietary recommendations for the prevention and management of nonalcoholic fatty liver disease in adults
Ratziu et al. (44)	2019	CINAHL	Expert opinion	Recommendations for management and treatment of nonalcoholic steatohepatitis
Younossi et al. (45)	2021	BMJ	Expert opinion	AGA clinical practice update on lifestyle modification using diet and exercise to achieve weight loss in the management of nonalcoholic fatty liver disease

Systematic review^*^: systematic evaluation and meta-analysis; Systematic review^**^: meta-analysis.

### 3.3 Results of the evaluation of the quality of literature

#### 3.3.1 Quality evaluation of guidelines

This review included seven guidelines (22–28). The average percentage and recommended grade for each domain are listed in Table 3. One guideline (24) had a final score percentage of ≥60% on all six domains and a recommendation grade of A. The remaining guidelines (22, 23, 25–29) had a recommendation grade of B. Seven guidelines were of good overall quality and approved for inclusion in the review.

**Table 3:** Quality evaluation of guidelines.

Included articles	The average percentage in each domain (%)						Comprehensive score 1 (scores)	Comprehensive score 2 (scores)	Number of domains with average scores ≥60% (number)	Number of domains with average scores ≥30% (number)	Recommended grade
	Range and purpose	Participants	Development rigor	Expression clarity	Applicability	Independence of editors					
Cusi et al. (24)	100	63.89	90.63	91.67	75.00	83.33	6.00	6.00	6	6	A
Eslam et al. (25)	97.22	55.56	41.67	94.44	83.33	66.67	6.00	4.00	4	6	B
Kang et al. (26)	94.44	58.33	69.79	94.44	52.08	66.67	5.00	6.00	4	6	B
AISF et al. (22)	88.89	36.11	88.33	88.89	72.97	70.83	6.00	5.00	5	6	B
Aller et al. (23)	86.11	41.67	44.79	88.89	75.00	58.33	5.00	5.00	3	6	B
Fouad et al. (28)	88.89	47.22	34.38	91.67	79.17	75.00	4.00	4.00	4	6	B
Chinese Society of Hepatology et al. (27)	77.78	41.67	29.17	80.56	25.00	0.00	4.00	4.00	2	3	B

#### 3.3.2 Quality evaluation of systematic reviews

A total of 9 systematic reviews (29–37) were evaluated using the AMSTAR 2 scale (16). One systematic review (34) had “yes” or “partially yes” responses for all seven key items and was evaluated with a grade of “medium.” Two systematic reviews had a response of “no” for 1 key item [item 13 for the study by Garcêz et al. (33) and item 7 for the study by Babu et al. (29)] and ≥1 non-key item, with an evaluation grade of “low.” The remaining six systematic reviews (30–32, 35–37) had a response of “no” for ≥1 key item and ≥1 non-key item, with an evaluation grade of “very low.” Nevertheless, since their “no” responses constituted less than 50% or “unclear” responses were less than 80% of the total evaluation items. Eventually, nine systematic reviews with relatively complete study designs were eventually analyzed.

#### 3.3.3 Quality evaluation of expert consensus and expert opinions

A total of six expert consensuses (38–43) and four expert opinions (44–47) were included in this review. In the evaluation of one expert consensus (46), the response to the second item, “Is the opinion derived from an influential expert in the field,” was “unclear,” while the responses to the remaining items were “yes.” The remaining nine publications had a “yes” response to all items. All expert consensus and expert opinion literature were of high quality and, hence, were deemed eligible for inclusion.

### 3.4 Summary of evidence

Following extraction, summarization, and analysis of the evidence related to lifestyle interventions for adults with MASLD, a total of 28 articles were finally analyzed. The most compelling evidence across six key dimensions was synthesized. They were intervention modalities, diet management, exercise management, weight loss management, personalized management, and multidisciplinary collaboration (Table 4).

**Table 4:** Best evidence of lifestyle interventions in adult patients with metabolic-associated fatty liver disease.

Types	Evidence content	Evidence level	Recommended grade
Intervention modalities	1. Lifestyle interventions are recommended for all adults with MASLD (regardless of being overweight or underweight) (22, 23, 26, 28, 34, 40, 43–44) to achieve weight loss through diet and exercise. Lifestyle interventions through structured programs are recommended.	A^①^	1
	2. Weight loss (through lifestyle changes or bariatric surgery) is recommended as the treatment of choice for adults with MASLD who are overweight or obese (especially abdominal obesity) (23, 39).	A	1
	3. Clinicians or nurses are recommended to encourage and educate adult patients with MASLD to change their poor lifestyles (26) and provide patients with guidance on lifestyle management (22), including healthy diet, increased physical activity and exercise, weight loss, and modification of poor lifestyle habits.	C	2
Diet management	4. Restriction of total calorie intake (23, 25–28, 35, 41, 44) and control of food intake are key to diet interventions in adults with MASLD (26). It is recommended to consume 1,500–1,800 kcal calories per day for men and 1,200–1,500 kcal calories per day for women or reduce the total calorie intake of the current diet by 500–1,000 kcal per day (25, 26, 28, 39, 45).	A	1
	5. Low-carbohydrate (30, 41) and low-fat diets are recommended for adults with MASLD.	B	1
	6. Adults with MASLD are recommended to follow the Mediterranean diet (22, 25, 26, 28, 41, 45, 47).	B	1
	7. The Japanese diet model (47), Dietary Approaches to Stop Hypertension (38), and the National Cholesterol Education Program (35) are beneficial for adults with MASLD. The ketogenic diet is not yet recommended for adults with MASLD (39).	5c^②^	B
	8. It is recommended that adults with MASLD adjust their diet to reduce the amount of macronutrients (such as saturated fat, starch, and added sugars) (24, 27) and increase the intake of dietary fiber (41, 47).	A	1
	9. Adults with MASLD are recommended to avoid deeply processed foods, fast foods, and snacks (25, 42, 44, 46) and replace them with fiber-rich unprocessed foods (such as whole grains, vegetables, fruits, legumes, nuts, and seeds) (46–47).	B	1
	10. It is recommended that adults with MASLD limit excessive fructose intake and avoid processed foods and beverages with added fructose (28, 46–47). It is recommended that industrial fructose intake should be limited to less than 5% of total daily carbohydrate intake (44).	B	1
	11. Caffeinated coffee is recommended for adults with MASLD (23, 27–28, 41, 44), and there are no data on the recommended dose of coffee (23).	A	2
	12. Alcohol consumption should be limited in adults with MASLD (27, 45–47), and abstinence from alcohol is recommended for adults with MASLD who have steatohepatitis or liver fibrosis (22–23, 41, 44).	B	1
	13. Alcohol may be a factor in the progression of liver disease for MASLD adults, and the intake of alcohol in MASLD adults should be assessed regularly (41).	5b	B
Exercise management	14. Exercise (with or without weight loss) is effective in reducing liver fat content (22, 24, 29, 42–44). Therefore, adults with MASLD are recommended to exercise regularly (45, 47) and participate in structured exercise programs whenever possible (24).	A	1
	15. Adults with MASLD are encouraged to increase their physical activity level as much as possible (22, 41) and to reduce sedentary behaviors (22).	A	1
	16. Both aerobic and resistance exercise are effective in decreasing liver fat content (24–25, 27–28, 30, 32, 36–37). Accordingly, adult patients with MASLD are advised to choose their preferred form of exercise that can be maintained over time (25, 27).	A	1
	17. Resistance exercise is recommended for adults with MASLD who have poor cardiorespiratory function or are unable to tolerate aerobic exercise, with better feasibility (25–26, 28).	B	2
	18. Adult patients with MASLD are recommended to engage in moderate or vigorous^*^ physical activity, 30 min or more per time and at least 3 times per week (24–26, 28) for at least 6 weeks (24). Brisk walking with no less than 150 min per week is recommended (23, 39, 45).	B	1
	19. Younger adults with MASLD may gain greater benefit from exercise than older adults with MASLD (34).	B	2
Weight loss management	20. It is recommended that all adults with MASLD (whether obese or not) who lose weight through diet and exercise can benefit from weight loss (23, 25, 28, 43).	A	1
	21. A structured weight loss program is recommended for overweight adult patients with MASLD (24).	B	1
	22. The following weight loss goals are recommended: 3–5% weight loss in non-obese patients with MASLD (23–24, 27) and 7–10% weight loss in patients with MASLD who are overweight or obese (23–26, 28, 38). Ideally, weight loss should exceed 10% (24, 44) with a weight loss of 0.5–1.0 kg per week (44).	A	1
	23. Bariatric surgery may be considered in MASLD adults with obesity or well compensated steatohepatitis and liver fibrosis who do not respond to lifestyle interventions and pharmacotherapy (26–28, 40–43).	B	2
Personalized management	24. It is recommended that the optimal daily calorie intake be adjusted according to the age, sex, weight, and physical activity of adults with MASLD (26).	5b	A
	25. It is recommended to tailor individualized diets (23, 26, 28, 38, 42, 44) to the diet needs and preferences of adult MASLD patients by nutritional experts whenever possible to achieve long-term patient compliance (26, 38)	A	1
	26. The choice of exercise needs to be individualized (41). It is recommended that exercise programs be tailored to the individual lifestyle and preferences of adults with MASLD whenever possible (24, 38).	5b	A
	27. The recommendation to offer bariatric surgery to MASLD adults with cirrhosis should be individualized (28) because of the high risk of postoperative complications.	C	1
Multidisciplinary collaboration	28. The establishment of a multidisciplinary team of experts including hepatologists, nurses, clinical nutritionists, exercise therapists, behavioral medicine specialists, and psychologists (25, 27, 38–39, 41, 47) is essential to increase the motivation and long-term adherence of patients with MASLD to participate in lifestyle interventions.	5b	A

^①The GRADE system was used for evidence grading; ②The 2014 version of the JBI evidence pre-grading and evidence recommendation level system was utilized.^

* Intensity of physical activity: moderate physical activity includes brisk walking, dancing, gardening, slow bicycling, and carrying objects <20 kg. Vigorous physical activities include running, fast uphill walking/climbing, fast bicycling, aerobics, fast swimming, and carrying objects ≥20 kg.

## 4 Discussion

### 4.1 Adults with MASLD can benefit from lifestyle modification interventions

The evidence summary revealed that lifestyle interventions (diet, exercise, and weight loss) were effective in reducing lipids, liver enzymes, intrahepatic fat content, and insulin resistance in patients (22, 26). Whilst there is emerging evidence for time-restricted eating, the impact of the time spent on diet and exercise is largely unknown. There is no clear consensus on the optimal diet regimen for adults with MASLD, such as calorie intake, diet pattern and structure, and the distribution of dietary components (ratio of carbohydrates, proteins, and fats, and ratio of saturated to unsaturated fats) (24, 44).

There is still debate about the frequency, intensity, duration, and type of exercise (25, 36). Furthermore, the protocols for aerobic exercise were consistent with the recommendations of the American College of Sports Medicine (36) in only 35% of studies. Therefore, large, multidisciplinary randomized controlled trials with adequate follow-up and robust documentation of diet and exercise adherence (49) are required to determine the optimal diet regimen and exercise prescription for adults with MASLD and to evaluate the health benefits of optimal diet and exercise doses for such patients (36).

All adults with MASLD can benefit from losing weight through changes in diet and exercise (45). However, the metabolic health of patients with MASLD benefits differently depending on the ratio of weight loss to the initial body weight. For instance, a weight loss of ≥5% can reduce hepatic steatosis, weight loss of ≥7% can contribute to the regression of steatohepatitis, and weight loss of ≥10% can lead to the regression or stabilization of liver fibrosis (5, 6, 13, 36, 37, 39). Furthermore, the type, safety, and efficacy of bariatric surgery for patients with MASLD are unclear and require careful benefit–risk assessment by a multidisciplinary team of experts, including hepatologists (41).

### 4.2 Adults with MASLD can benefit from the development of structured lifestyle intervention programs

Several guidelines and consensus statements recommend lifestyle modification interventions through structured programs for adults with MASLD, especially for patients with advanced liver fibrosis and/or at high risk of rapid fibrosis progression (38). Syntheses of the available evidence revealed that there is no uniform, standardized, and structured lifestyle intervention program for adults with MASLD.

The purpose of this evidence summary was to provide a foundation for the development of standardized and structured lifestyle intervention programs for adults with MASLD by focusing on the evidence related to diet, exercise, and weight loss, thereby helping medical staff and healthcare professionals efficiently acquire and understand the evidence for such interventions. Future research should focus on the development and use of localized and structured lifestyle intervention programs based on the diet, exercise habits, and cultural norms of adults with MASLD.

### 4.3 Lifestyle intervention programs for adults with MASLD should be individualized as much as possible

Evidence shows that adults with MASLD have poor long-term compliance with lifestyle changes (44) and struggle to sustain healthy diet habits in the long-term (26). More than 80% of patients with MASLD do not complete a physical activity program of 30 min of moderate-intensity exercise per session and ≥3 times per week (36). It is equally challenging to achieve and maintain weight loss (28, 41). In a study of a 52-week structured weight loss intervention, only 30% (88/261 cases) of patients with MASLD achieved a weight loss of ≥5% (50). Only 32% of patients with MASLD who were overweight or obese achieved a weight loss of ≥5% during a 5-year follow-up, and among them, only 25% maintained their weight loss 5 years after the intervention (48). Therefore, several guidelines and consensus statements recommend that individualized lifestyle intervention programs be tailored to the culture, personal preferences, and needs of patients with MASLD to improve intervention outcomes and promote long-term adherence and compliance with the intervention program (38, 41).

### 4.4 A multidisciplinary team can increase the motivation of adults with MASLD to participate in and comply with lifestyle interventions

Although adherence to lifestyle changes can benefit patients with MASLD, research indicates that long-term compliance is poor in most patients during clinical implementation (44). To effectively design and implement structured lifestyle intervention programs for adults with MASLD, it is imperative to assemble a multidisciplinary team of experts. This team should ideally include hepatologists, nurses, clinical nutritionists, exercise therapists, behavioral medicine specialists, and psychologists. Additionally, studying the impact of various intervention models on long-term compliance and intervention outcomes is essential for the development of successful interventions. It is important that this team collaborate with patients and their support systems to develop structured and individualized lifestyle intervention programs incorporating different combinations of strategies (41). This method can be effective in managing lifestyle interventions for adults with MASLD as it increases their motivation and compliance (38).

## 5 Conclusion

Lifestyle intervention is a safe, low-cost, and highly effective method that is the foundation of treatment for MASLD. Nevertheless, the clinical application of lifestyle interventions remains suboptimal, and there is a significant issue with poor compliance in real-world clinical practice. In this review, we summarized the best evidence on lifestyle interventions for adults with MASLD by systematically identifying high-level evidence-based information from China and elsewhere and provided practice recommendations in six areas: intervention modalities, diet management, exercise management, weight loss management, personalized management, and multidisciplinary collaboration.

Through this review, we aimed to encourage clinical staff and healthcare professionals to follow the best evidence and guide adults with MASLD toward adopting standardized interventions for a healthy lifestyle. The use of evidence related to lifestyle interventions should be integrated with and tailored to suit the sociocultural situation of adults with MASLD, such as personal preferences, lifestyle norms, and behavioral habits, while keeping in mind the feasibility and applicability of the evidence. This can increase the compliance of patients and promote the translational application of the evidence. The evidence summary presented here requires continuous updates with the inclusion of newly published literature. Furthermore, there is a need for further studies to explore the utilization of the best available evidence in designing effective lifestyle modification interventions tailored for adults diagnosed with MASLD.

## Data Availability

The original contributions presented in the study are included in the article/supplementary material, further inquiries can be directed to the corresponding authors.
